# Analysis on the Possibility of Eliminating Interference from Paraseismic Vibration Signals Induced by the Detonation of Explosive Materials

**DOI:** 10.3390/s20216401

**Published:** 2020-11-09

**Authors:** Józef Pyra, Maciej Kłaczyński, Rafał Burdzik

**Affiliations:** 1 Department of Mining Engineering & Occupational Safety, Faculty of Mining & Geoengineering, AGH University of Science and Technology, Mickiewicza 30, 30-059 Cracow, Poland; 2 Department of Mechanics and Vibroacoustics, Faculty of Mechanical Engineering and Robotics, AGH University of Science and Technology, Mickiewicza 30, 30-059 Cracow, Poland; maciej.klaczynski@agh.edu.pl; 3 Department of Automotive Vehicle Construction, Faculty of Transport and Aviation Engineering, Silesian University of Technology, 40-019 Katowice, Poland; rafal.burdzik@polsl.pl

**Keywords:** structural vibrations, acoustic wave, airblast shock wave, signal filtering, geophone

## Abstract

This article presents the results of studies on the impact of acoustic waves on geophones and microphones used to measure airblasts carried out in a reverberation chamber. During the tests, a number of test signals were generated, of which two are presented in this article: frequency-modulated sine (sine sweep) waves in the 30–300 Hz range, and the result of detonating 3 g of pyrotechnic material inside the chamber. Then, based on the short-time Fourier transform, the spectral subtraction method was used to remove unwanted disruption interfering with the recorded signal. Using MATLAB software, a program was written that was calibrated and adapted to the specifics of the measuring equipment based on the collected test results. As a result, it was possible to clean the signals of interference and obtain a vibration signal propagated by the substrate. The results are based on signals registered in the laboratory and made in field conditions during the detonation of explosive materials.

## 1. Introduction

Ground vibrations, building vibrations, shock waves (airblast waves) and acoustic waves are mechanical waves and, therefore, propagate in elastic media. When under the influence of a one-time applied force in the examined medium and moving elastic vibrations of its particles arise, it can be assumed that in this environment, an elastic wave running at finite speed is created. Then, after the disturbance-causing impact disappears, every particle deviating from the equilibrium position will begin to return, with a simple vibrating motion with acceleration toward this point. This is what happens in the case of detonation of explosive materials, especially when they are detonated on the surface or under a large protective layer. The impact is propagated both through the ground, reaching buildings, as well as through the air in the form of air shock waves and acoustic waves [1,2]. If the air shock wave has lower pressure values (approx. 20–50 Pa), it is difficult to pinpoint exactly where it transforms into an acoustic wave. Among the first studies related to the acoustic aspects of blasting operations in mines were the works [1,3,4,5], in which it was noted that the acoustic wave can affect both buildings and the psychological comfort of people in buildings. In a study by Pegden et al. [6], it was found that vibrations recorded in the ground directly in the vicinity of the building were the cause of the occurrence of an acoustic reaction in the building, and airblast pressure did not affect it. Lusk et al. [7] stated that in the case when airblast was identified as the dominant noise source, the peak sound amplitude was directly related to the airblast pressure amplitude. However, when ground vibrations were identified as a source of noise in the object, the peak sound amplitude was not related to the ground vibration amplitude. In addition, they also reported that beyond a distance of 2500 ft (762 m), a greater sound wave is generated by ground vibrations, rather than by airblast pressure. In addition, the acoustic effect in buildings can be enhanced by the shaking of, e.g., glass caused by overpressure or ground vibrations, and thus cause vibrations in the building [6,7]. In the works of Margrave et al. and Steiner [8,9] the authors noted that when the frequency structure of a seismic signal is close to the frequency structure of an acoustic wave, the seismic signal is disturbed by noise.

When considering ground vibration propagation, a large amount of application can be found. For example, Kowalska-Koczwara et al. [10] presented the case of determining the vibration comfort of people in buildings above subway tunnels using WODL ratio (human vibration perception index). Moreover, high-speed rail ground vibrations are researched in many publications [11,12,13]. Thompson et al. [14] presented a general overview of the evaluation of ground vibration. In the paper review, evaluation criteria for both feelable vibration and ground-borne noise on railway vibration and noise are given. Romero et al. [15] presented a numerical method to study noise and vibration from underground structures. They described a model that represents the soil structure interaction problem and the radiated noise and vibration. The paper also presents developed applications to analyze the acoustic and elastic wave propagation from underground structures.

Gupta et al. [16] explained, however, that the vertical component of vibrations of a rock medium causes the creation of an equivalent sound speed. This causes vibrations in the air of the same frequency as the vertical vibrations of the rock medium, which can cause interference with seismic signals. Moreover, the authors of the following works [9,17,18,19,20] analyze the possibilities of reducing the noise transmitted by air from the vibrator baseplate to seismic recordings. For this purpose, they use microphones for recording sound, so that at a later stage these recordings can serve as a kind of “mask filter”, thanks to which it is possible to separate the seismic signal. In addition, the works [18,19] presented an analysis of a problem known since 1951, when it was first found out that an air-propagated wave can also exchange energy with existing surface waves of the substrate moving in the layer at the surface. As a result, an air-coupled surface wave is generated when the specific frequency component of the Rayleigh scattered wave is equal to the speed of sound in the air [21,22,23].

Wind noise reduction can be achieved from seismic signals by applying the methodology described in U.S. patents [24,25]. In addition, as described in the patents, the device only suppresses wind noise—its ability to suppress other types of air noise (e.g., airblast) is unknown [20]. The coexistence of different propagation paths of mechanical vibrations may cause a change in the recorded intensity of vibrations propagated by the substrate or air, which may translate into an erroneous assessment of the impact of vibrations on building structures [26,27]. This problem is very important, especially in the situation when impact measurements are carried out at a short distance from the place, e.g., during the detonation of explosive materials, and are to be used to assess their impact on buildings [28,29]. Therefore, the following sections of the article present examples of vibration measurements and recordings made by airblast and acoustic microphones during tests in the reverberation chamber of AGH UST in Krakow; then, the signals presented in an earlier work are filtered based on the authors’ developed model and program [30].

## 2. Sensor Diagnostics in Laboratory Conditions—Tests in the Reverberation Chamber

**Figure 1:**
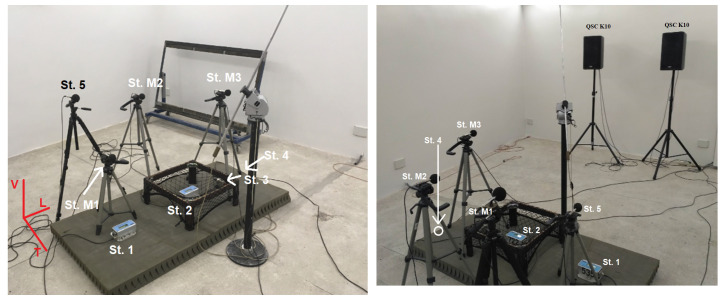
Picture of sensors in the reverberation chamber.

**Figure 2:**
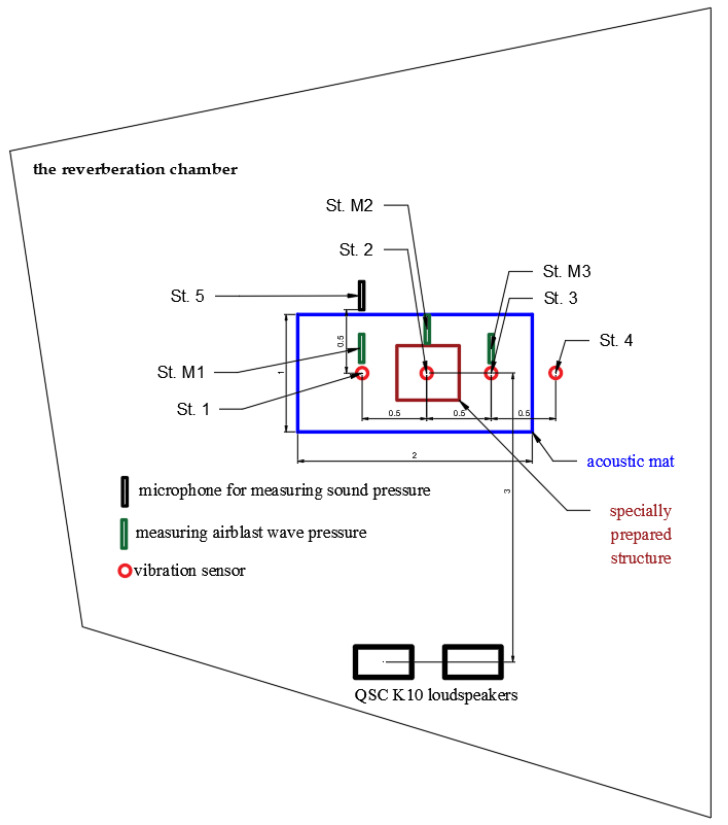
Distribution of sensors in the reverberation chamber.

Bearing in mind the examples of measurements presented in our earlier works [30], measurements were carried out in the reverberation chamber with the inclusion of an apparatus for measuring sound pressure levels in the 1 Hz–20 kHz band. The main purpose of this was to check the impact of the sound wave (recording with acoustic measurement microphones) on geophones and microphones used for airblast pressure measurement. In each case, a UVS 1608 apparatus by the Swedish company Nitro Consult AB or a VIBRALOC by the Swedish company ABEM Instrument AB was used for measurements, equipped with three-component geophones and broadband microphones, and in some measurements, SVAN 958 vibration and sound level meters and analyzers were used. The apparatus for measuring vibrations was positioned in such a way as to see the effect of the acoustic wave on the meter located on various substrates. The measuring stations are arranged as follows (All stations are marked in Figure 1 and Figure 2):

St. 1—the vibration sensor is placed on a double acoustic mat (Vibraloc SN 535);
St. M1—microphone for measuring airblast wave pressure directly above St. 1 (Vibraloc SN 535);
St. 2—sensor for measuring vibrations suspended on a specially prepared structure (Vibraloc SN 722);
St. M2—microphone for measuring the pressure of the airblast wave directly above St. 2 (Vibraloc SN 722);
St. 3—the vibration sensor is placed on a double acoustic mat (UVS 1608 SN 0048);
St. M3—microphone for measuring airblast wave pressure directly above St. 3 (UVS 1608 SN 0048);
St. 4—vibration sensor located directly on the floor (UVS 1608 SN 0048);
St. 5—microphone for measuring sound pressure (SVAN 958).

The directivity of the measurement at each measuring stand was consistent with the axes of the orthogonal reference system, so vibrations were recorded on the vertical axis and on two horizontal axes: longitudinal and transverse (Figure 1).

Using two active QSC K10 loudspeakers and a KEITHLEY 3390 generator generated a series of test signals which were sinus modulated (sine sweep) in the 30–300 Hz range. At the very end, in the reverberation chamber, the same pyrotechnic material was set off, but with a relatively lower mass (3 g) than during field tests at the testing ground (Figure 19 in [30]).

Two examples of tests were selected to illustrate the experiments:

No. I sine sweep, 30–300 Hz, maximum gain on the generator and speaker;
No. II: pyrotechnic material.

Sixteen measurements were made at different equipment settings (generator and speaker gain, frequency range, tone, and explosive). Two of them were selected for presentation in this publication. Records of vibrations and airblast wave pressure from individual measurement stations were presented as time waveforms and their frequency structure. The frequency structure is shown as an Fast Fourier Transform (FFT) amplitude spectrum plot (module plot).

**Figure 3:**
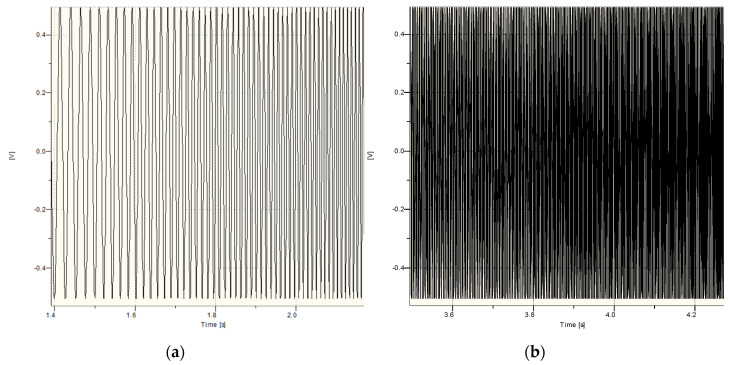
Time courses of sine sweep, 30–300 Hz range: beginning of the signal (**a**), end of the signal (**b**).

**Figure 4:**
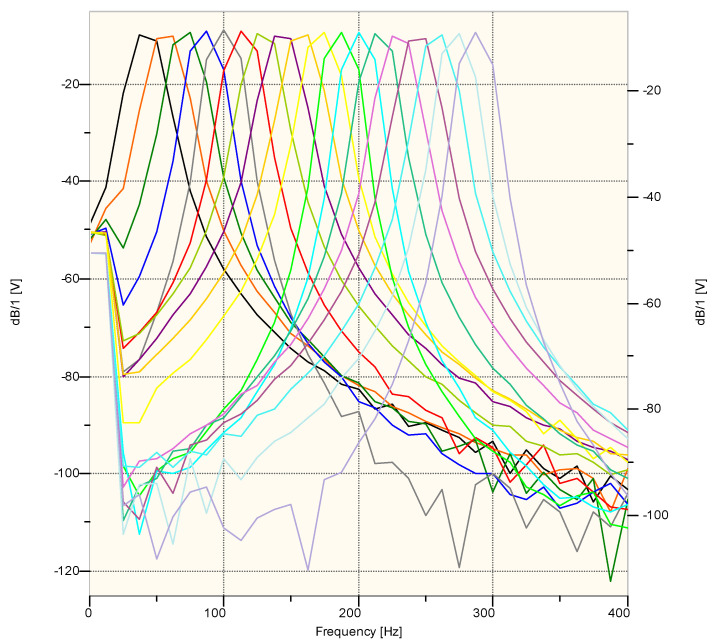
FFT analysis of sine sweep: 30–300 Hz; 3 s of signal; fs = 51,200 Hz; nfft = 4096 samples.

For the sine sweep signal, the frequency modulation time was 3 s for the 30–300 Hz range. Figure 3 show the time course from the generator, and Figure 4 show the FFT analysis of this signal (3 s of signal, fs = 51,200 Hz, FFT nfft = 4096 samples,). The tested range covered the frequency band in which the impact of the airblast wave on the ground vibration velocity sensor (geophone) was observed in our measurement practice, which is presented in [30].

**Figure 5:**
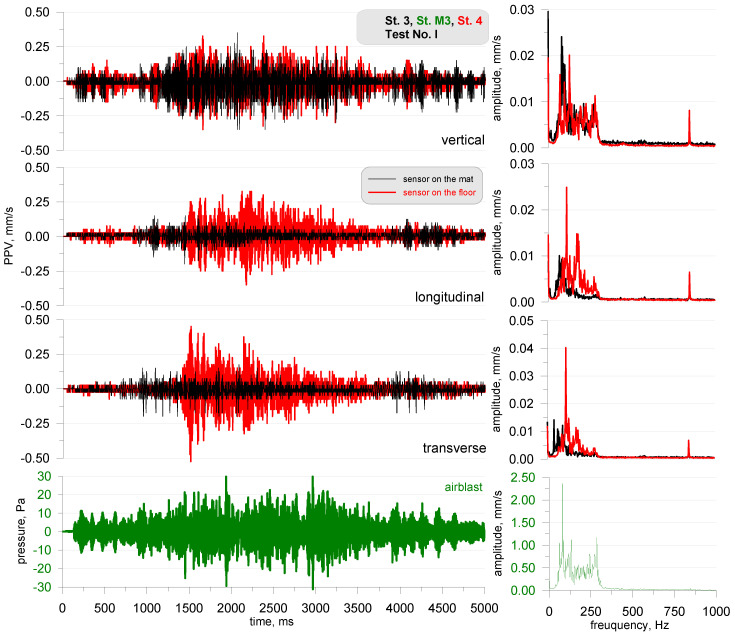
Test No. I: vibration seismogram (three directional components) and airblast pressure record with FFT analysis; sine sweep range of 30–300 Hz; stand 3 and 4.

Figure 5 presents the recordings from the sensor placed on the acoustic foam (black—stand 3) and on the floor (red—degree 4) for test No. I. The sensors behaved in the same way as in the previous test, i.e., a comparable vibration level for the vertical component and a higher vibration level for the horizontal components for the sensor located directly on the chamber floor were observed. The frequency structure corresponded to the range of the excited acoustic wave. The pressure level recorded by the microphone for measuring the airblast wave did not exceed 30 dB for either test.

**Figure 6:**
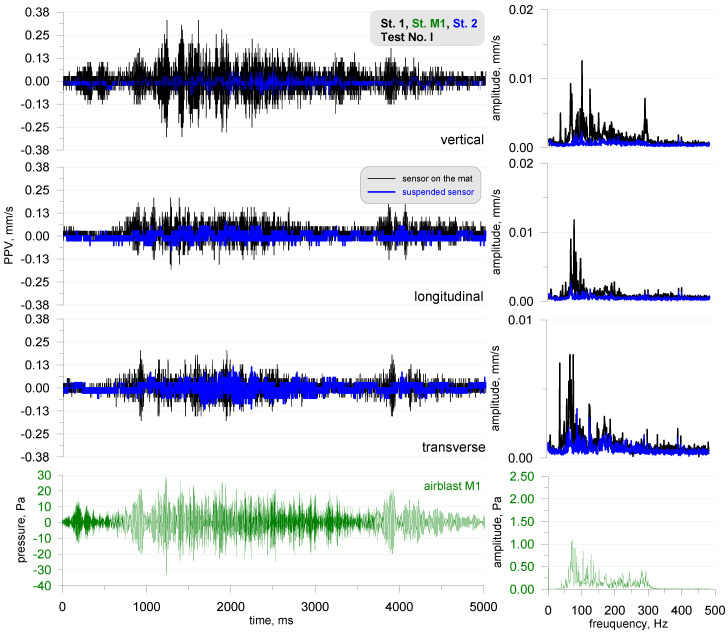
Test No. I: vibration seismogram (three directional components) and airblast pressure record with FFT analysis; sine sweep in the range of 30–300 Hz; stand 1 and 2.

The next figure (Figure 6) illustrates the results of the recording of the vibration and airblast pressure wave recorded at stands 1 and 2 for test No. I. The black line is the measurement from the sensor placed on the mat, and the blue line is the sensor suspended on the structure. It can be seen that the vibrations registered by the suspended sensor are smaller than those registered by the sensor placed on the acoustic foam. The disturbance that was recorded by the suspended sensor would not have triggered the measurement recording were it not for the connected microphone for measuring the airblast pressure. Analyzing the frequency structure, it was apparent that from the suspended sensor signal, it is difficult to indicate the frequency range of recorded vibrations. There was no such clear distortion of frequencies that was excited by an acoustic signal.

**Figure 7:**
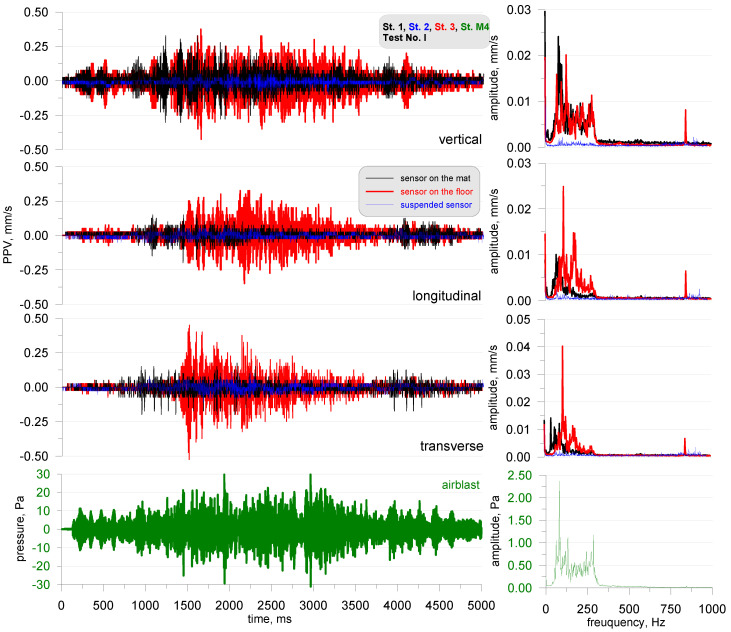
Test No. I: vibration seismogram (three directional components) and airblast pressure record with FFT analysis; sine sweep in the range of 30–300 Hz; stands 1, 2 and 4.

In order to illustrate the whole, in Figure 7, the entries from the stands are presented: 1—black line (sensor on the acoustic foam), 2—blue line (suspended sensor) and 4—red line (sensor on the floor of the chamber). The drawing depicts test No. I. The recording registered by the suspended sensor is characterized by the lowest values of vibration velocity in all three components. Analyzing the FFT result, it can be seen that for test No. I, the entire frequency range corresponding to the excited acoustic signal is apparently visible for the sensor placed on the chamber floor. However, the sensor placed on the mat did not register a frequency above 100 Hz (this can be clearly seen in Figure 5). The acoustic foam, therefore, dampened these higher frequencies. Suspension of the sensor also caused it to be isolated from the influence of these lower frequencies.

**Figure 8:**
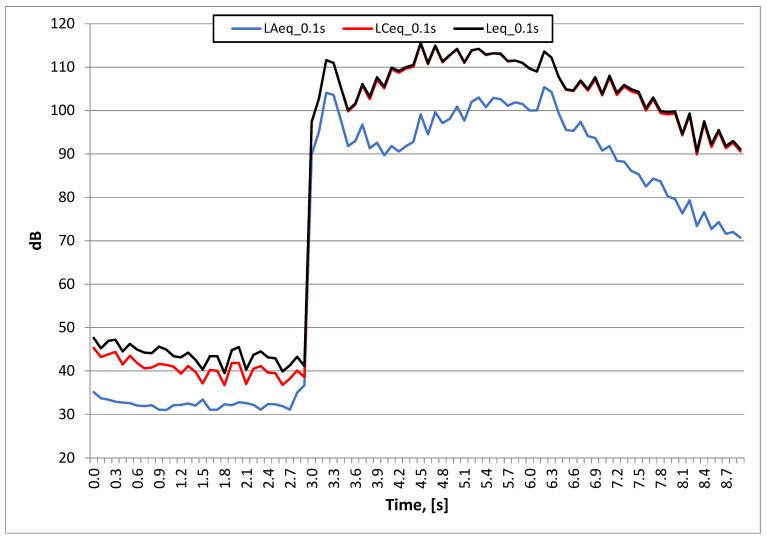
Test No. I: sound pressure level waveform sine sweep in the range of 30–300 Hz (ZZ17); stand 5.

**Figure 9:**
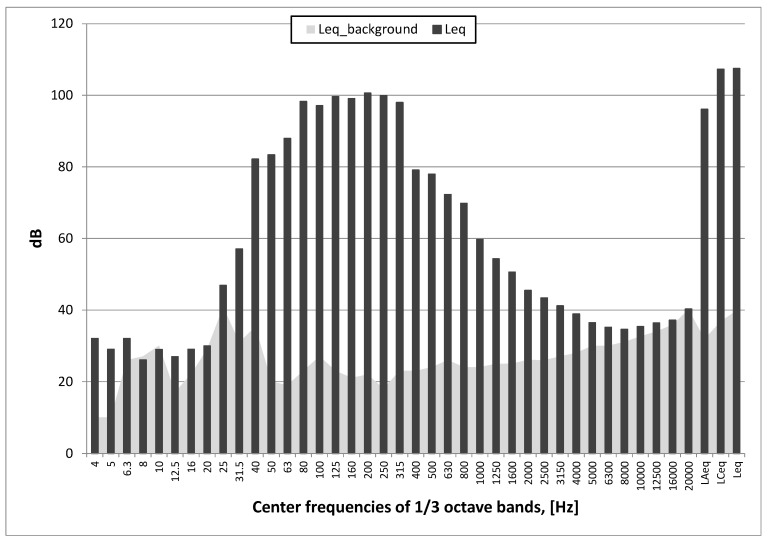
Test No. I: spectrum of averaged sound pressure levels in 1/3 octave bands; sine sweep in the range of 30–300 Hz and background noise (Leq_background); stand 5.

As can be seen in Figure 8 and Figure 9 (test No. I: sine sweep signal in the range of 30–300 Hz), the maximum unweighted and C-weighted sound pressure level was over 115 dB, while the maximum A-weighted sound pressure level was over 100 dB.

**Figure 10:**
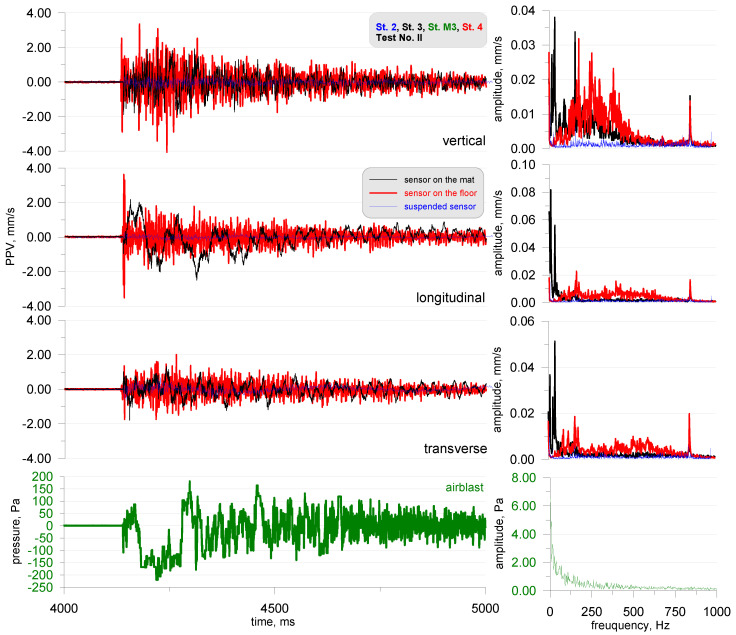
Test No. II: vibration seismogram (three directional components) and airblast pressure record together with FFT analysis; pyrotechnic material explosion; stand 1, 2 and 4.

As the last recording from this series of tests (Figure 10), the recordings from stands 1, 2 and 4 were induced by detonation of 3 g of pyrotechnic material placed on the chamber floor. In order to record everything, the apparatus was turned on earlier, so the recording related to detonation started from 4 and had the character of an impulse that disappeared after 1 s. Similar to the tests induced by the generated acoustic wave, the largest impact was registered by a sensor located directly on the chamber floor. Slightly lesser values were recorded by the sensor placed on the acoustic foam and the smallest values by the suspended sensor. Analyzing the records themselves and the frequency structure, it can be seen that the sensor placed on the floor had a very wide range from 1 to approx. 600 Hz. The sensor had a frequency range recorded only by the vertical component, while the horizontal components recorded the range from 1 to approx. 50 Hz. The suspended sensor lacked a dominant frequency range. In the frequency structure, all tests had a peak (local maximum value) at a frequency of approx. 800 Hz (Figure 5, Figure 7 and Figure 10); this did not occur for the suspended sensor (Figure 6). This is the resonance frequency (mode) of the reverberation chamber, mode 15 15 15, equal to 794 Hz. The maximum value of the airblast pressure was 220 Pa, much more than that obtained during the generation of the acoustic wave. This translated into the level of vibrations recorded by the sensors, where it exceeded 3 mm/s. This was greater compared to previous tests during which the value of 0.5 mm/s was not exceeded.

**Figure 11:**
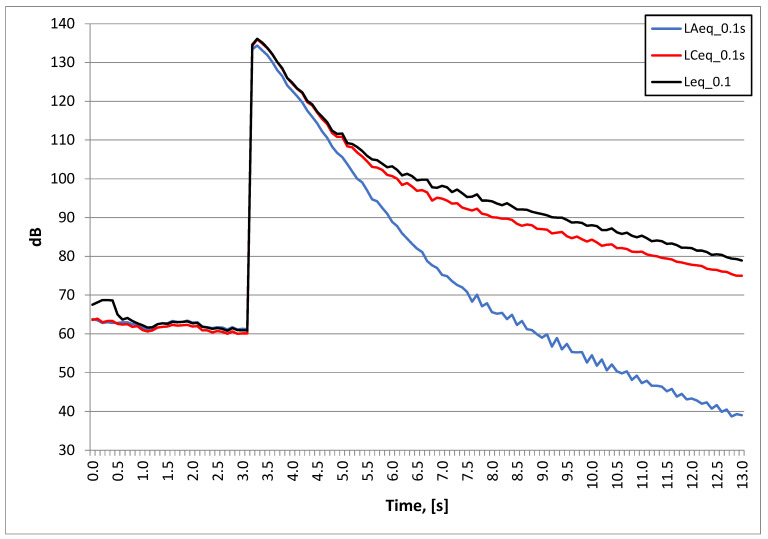
Test No. II: sound pressure level pyrotechnic material explosion; stand 5.

**Figure 12:**
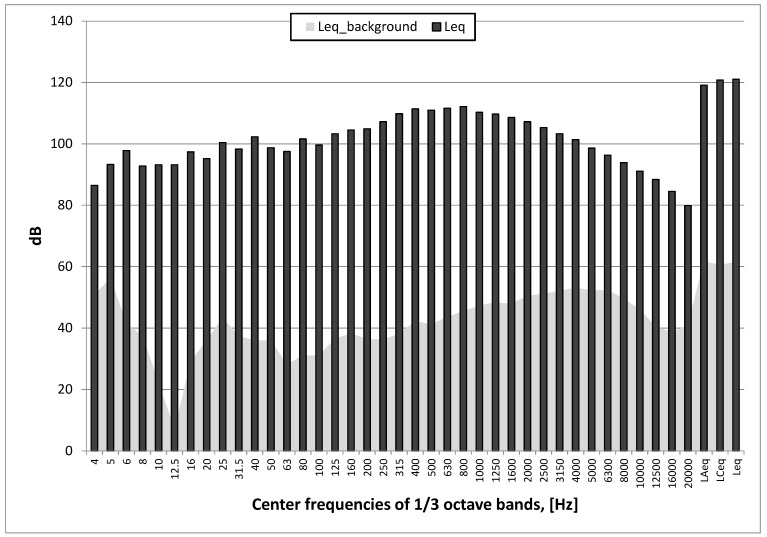
Test No. II: spectrum of maximum sound pressure level in 1/3 octave bands; pyrotechnic material explosion and background noise (Leq_background); stand 5.

As can be seen in Figure 11 and Figure 12 (pyrotechnic material explosion 3 g), the maximum level of unweighted and C-weighted sound pressure was over 135 dB, while the maximum level of A-weighted sound pressure was slightly lower and was 131 dB.

Summing up the results of the tests carried out in the reverberation chamber, it can be stated that the location of the geophone is very important for interference in the recording of vibration velocities. The geophone which was suspended and, therefore, the most isolated from the transmission of vibrations through the floor, recorded the lowest values of vibration velocities in all tests. The geophone placed on the mat recorded lower values than the sensor placed on the floor, but only for horizontal components, because the vertical component had similar values. In addition, it should be borne in mind that airborne acoustic vibrations may give rise to structural acoustic waves. Analyzing the above cases, it can be presumed that as a result of the generated sound wave (a generator with speakers), a structured acoustic wave propagated by the floor was created, which was recorded by the sensors. Therefore, the highest values of vibration velocity were recorded by the geophone located directly on the floor, slightly lower values on the mat, and practically no registration on the suspended sensor. The same phenomenon probably occurred in the case of the pyrotechnic material detonation at the training ground and recording of vibrations on the internal and external wall of a building (Figure 19 from [30]). The air acoustic wave caused the formation of a structural wave in the ground and in the structure of the object; then, as a result of wave penetration, the air acoustic wave inside the object was again formed. This could cause more interference on the internal sensor than the external sensor.

The loudspeakers and the acoustic wave generator used did not cause pressure excitation greater than 30 Pa recorded by the airblast microphone, and for the sound wave, the amplitude was about 11 Pa, despite the use of the maximum capabilities of the equipment used. Detonation of 3 g of pyrotechnic material caused a pressure of 220 Pa of airblast wave, and for the sound wave, the amplitude was about 112 Pa, which led to greater impact on the vibration sensors. Thus, it can be concluded that a significant change in pressure, which accompanies the airblast wave, is required for a significant impact on vibration sensors. Noise alone, even with very high sound pressure but not a sudden increase, does not cause a large impact on the sensor itself, but it can cause the formation of a structural acoustic wave that will be recorded by geophones, and thus will cause signal interference that will not be mechanical vibrations propagated through the ground.

## 3. Mathematical Model and Identification of Signal Elements

In order to better characterize the structure of the recorded signals, it is possible to perform a number of analyses ranging from time domain analysis, passing through spectral analysis, signal filtering, short-time Fourier transform, wavelet transform and ending with the matching pursuit algorithm [31,32,33,34,35]. On the basis of measurements, tests and observations made, a mathematical model of filtration of recorded signals has been developed, whose task is to use recordings from the airblast microphone to filter out interference on recordings from geophones.

For signal analysis in the frequency domain, so-called spectral methods are many times more effective in analysis than time methods. By using frequency language tools, the character of the signal can often be presented in a much simpler and illustrative way [36]. There are many techniques for frequency analysis. Despite the substantial technological progress that is still taking place, the basic Fourier transformation is the basic tool for such analyses. The spectral subtraction method is one of the oldest and longest-existing interference clearing and signal reconstruction solutions for speech, among other uses [37]. It is assumed that this method was created in 1979, when Steven F. Boll developed the formula on which it is based. Although it has existed for a long time, it is still an inspiration for new research, confirming its effectiveness. Nowadays, it is used in utility programs, while in the literature, there are numerous developments of the algorithm on which this method is based [38].

Spectral subtraction is a method that allows for the removal of unwanted disruption interfering with the signal. After performing the FFT analysis and then subtracting the adapted noise from the disrupted signal, a useful signal is created [39]. By transforming the previous expression, where the estimated signal is searched variable, we finally obtain the spectral subtraction Formula (1):

(1) $${\left|\hat{S}\left(e^{j\omega }\right)\right|}^{2}={\left|D\left(e^{j\omega }\right)\right|}^{2}-\alpha {\left|\hat{N}\left(e^{j\omega }\right)\right|}^{2}$$

where

$D\left(e^{j\omega }\right)$—spectrum of the signal with an interference (spectrum recorded by a geophone);
$\hat{S}\left(e^{j\omega }\right)$—estimate of the usable signal (signal after filtration);
$\hat{N}\left(e^{j\omega }\right)$—spectrum of the interference signal (signal from the airblast microphone);
α—interference factor in the noisy signal.

The above formula arises from a simple sum of components that are subjected to FFT. The α parameter allows for determining the impact of interference levels on the recorded signal, i.e., the impact of the path and propagation conditions. For stationary and ergodic interfering signals (noise), the de-noise operation takes place for one fragment of the interfering signal. However, in the case of a non-stationary interference signal, an analysis should be performed based on the STFT (short-time Fourier transform). The study assumed the length of the FFT analysis time frame, as equal to FTL = 250 ms (Frame Time Length).

In order to obtain the spectrum profile of the interference signal $\hat{N}\left(e^{j\omega }\right)$, a signal from the airblast microphone recording was taken for a given time frame. The real part of a complex number (*Re*) and imaginary part (*Im*) resulting from the STFT transformation are used to present the noisy signal—separately for both the real part (2) and for the imaginary part (3).

(2) $${\left|{\hat{S}}_{Re}\right|}^{2}={\left|D_{Re}\right|}^{2}-\alpha {\left|{\hat{N}}_{Re}\right|}^{2}$$

(3) $${\left|{\hat{S}}_{Re}\right|}^{2}={\left|D_{Re}\right|}^{2}-\alpha {\left|{\hat{N}}_{Re}\right|}^{2}$$

Division into two formulas means that each analyzed fragment of the noisy signal, as well as the built noise profile (in a given time frame), has its real values *Re* and imaginary values *Im*. Subtraction is, therefore, performed separately on both components in a given time frame. After the two-component (*Re* and *Im*) reduction, the de-noised signal is converted back into a time domain.

The presented mathematical model was implemented in a MATLAB environment to perform seismic signal filtering. The results of its application are presented in subsequent figures, and were made on the signals presented in earlier drawings.

**Figure 13:**
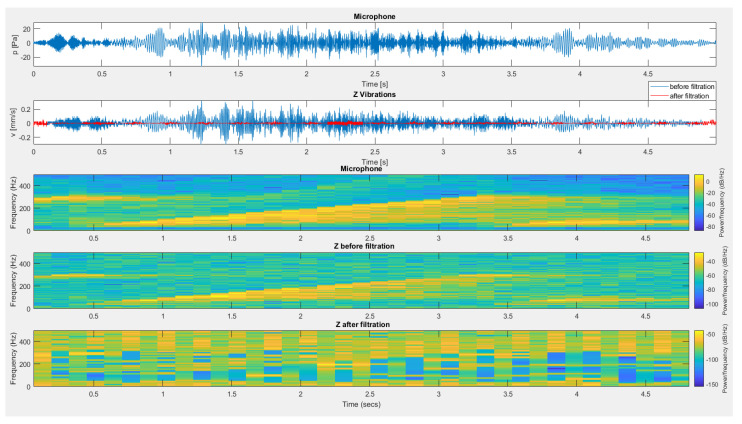
The result of using filtration for the data from the recording shown in Figure 5 (test No. I) for the vertical component of the sensor placed on the mat.

**Figure 14:**
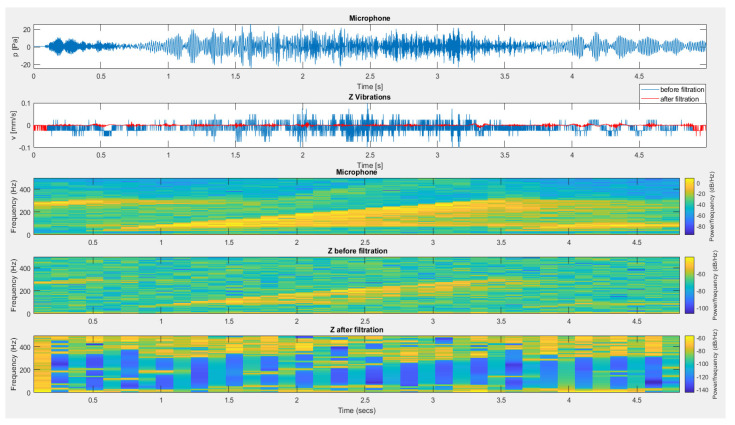
The result of using filtration for the data from the recording shown in Figure 6 (test No. I) for the vertical component of a suspended sensor on the structure.

**Figure 15:**
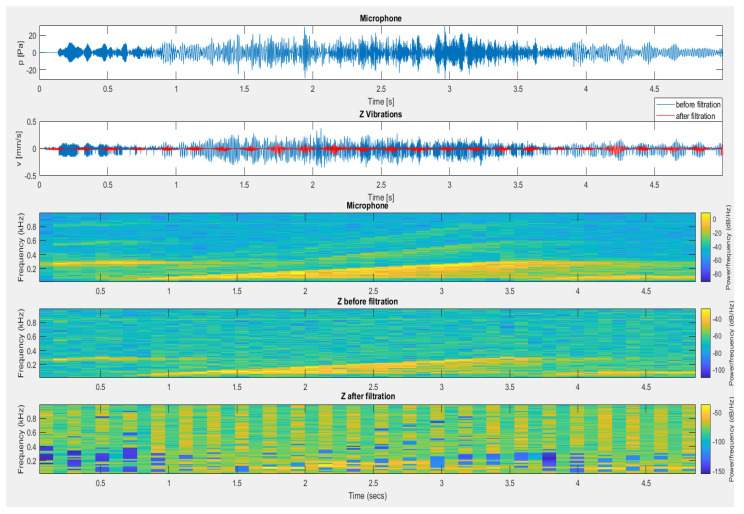
The result of using filtration for the data from the recording shown in Figure 7 (test No. I) for the vertical component from a sensor located directly on the floor.

Figure 13, Figure 14 and Figure 15 present the effect of filtering for the vertical component of vibrations recorded during test No. I for the sensor placed on the mat (Figure 13), the sensor suspended on the structure (Figure 14) and the sensor placed directly on floor (Figure 15). In all cases, the use of filtration removed interference from the geophone signal caused by the material acoustic wave resulting from the generation of an air acoustic wave.

**Figure 16:**
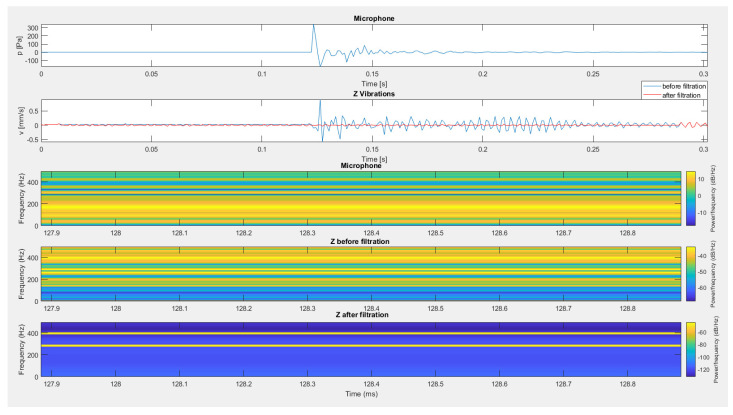
The result of using filtration for a vertical component from a sensor located outside the building.

**Figure 17:**
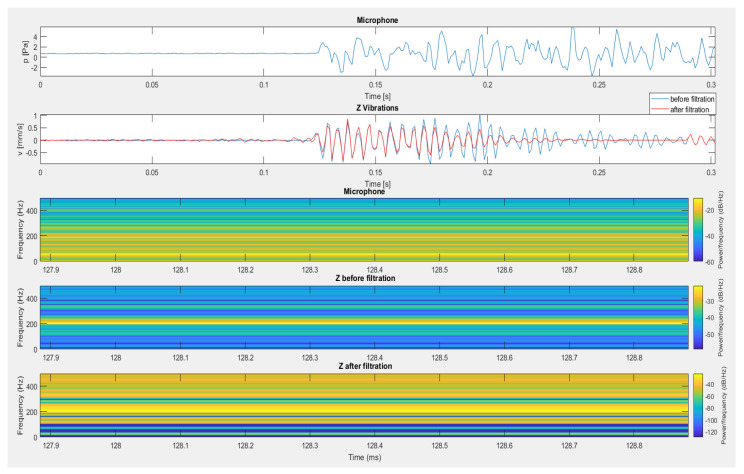
The result of using filtration for the vertical component from a sensor placed inside the building—an internal microphone.

The next recording subjected to the filtration process was the recording of vibrations registered on the load-bearing wall of the building by sensors placed outside (Figure 16) and inside (Figure 17) of this structure during the detonation of 3 g of pyrotechnic charge suspended at a height of 0.6 m. Interference generated outside the range of filtration resulted in the complete removal from the recording of vibrations of interference caused by impulse noise.

**Figure 18:**
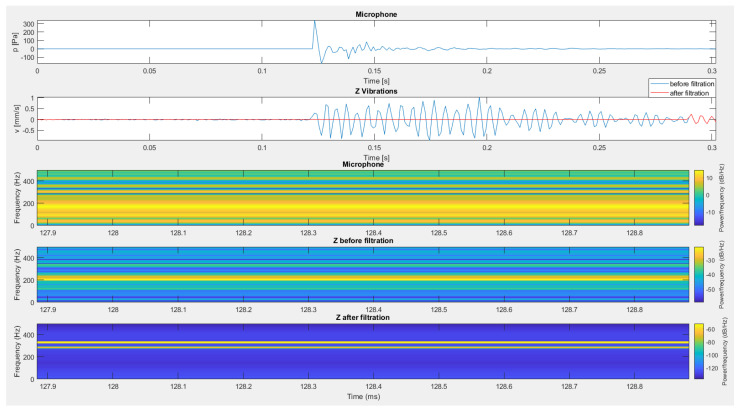
The result of using filtering for the vertical component from a sensor located inside the building—an external microphone.

The situation is slightly different in the case of a sensor placed inside the building structure (Figure 17). Here, the use of a signal from the microphone placed inside the building structure as a specific filter did not cause significant changes in the recording made by the geophone. This is probably due to the fact that the frequency structure of both signals is significantly different (Figure 19 from [30]). The frequency structure of the signal from the airblast microphone placed inside the building is dominated by higher frequencies that are not found in the geophone signal. In order to verify this, a recording from the airblast microphone placed outside the building was used to filter the signal from the sensor placed inside the building (Figure 18).

The use of recording from an external microphone for filtration removed the interference from the internal sensor. The acoustic wave created inside the building was modified so much that its recording did not correlate with the sensor recording for vibration measurement. The use of recording from an external microphone in both sensors (external and internal) removed interference caused by an acoustic wave.

**Figure 19:**
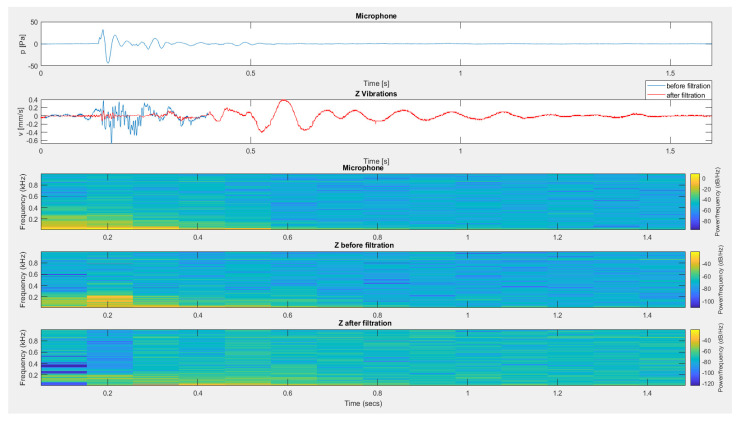
The result of using filtration for the vertical component from a sensor located outside the building—detonation of dynamite material placed in a short blast hole.

**Figure 20:**
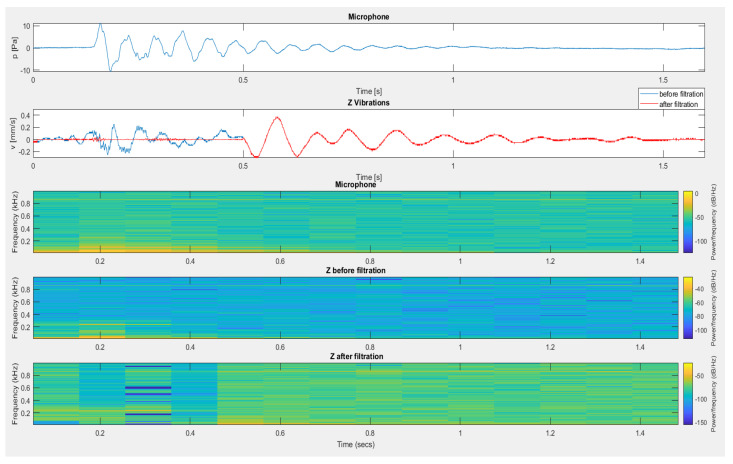
The result of using filtration for the vertical component from a sensor placed inside the building—detonation of dynamite material placed in a short blast hole.

Figure 19 and Figure 20 present the results of filtering for the vibration and airblast pressure signals induced by the detonation of a dynamite charge placed in a short blast hole. Figure 19 illustrates the filtering effect for a sensor placed outdoors on a load-bearing wall using a microphone signal placed outside. In contrast, Figure 20 shows the filtering effect of a sensor placed inside a building using signal from a microphone placed inside the building. In both cases, the recorded fragment caused by airblast impact on the geophones was removed from the signal on the vibration sensors.

**Figure 21:**
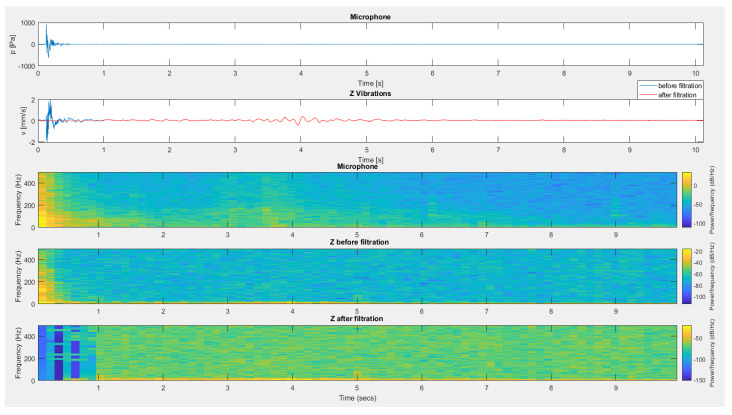
The result of using filtration for the vertical component—testing the fall of a structure to the ground.

Figure 21 shows the filtration effect for signals recorded at the time of setting off of the cumulative explosive material at a significant height. The registered impact came only from the action of a very high airblast pressure, which, at the same time, acted on the microphone and vibration sensor. The fall of the steel structure onto the ground was recorded later in the signal from the vibration sensor and was not filtered (recording at approx. 4 s—red line).

**Figure 22:**
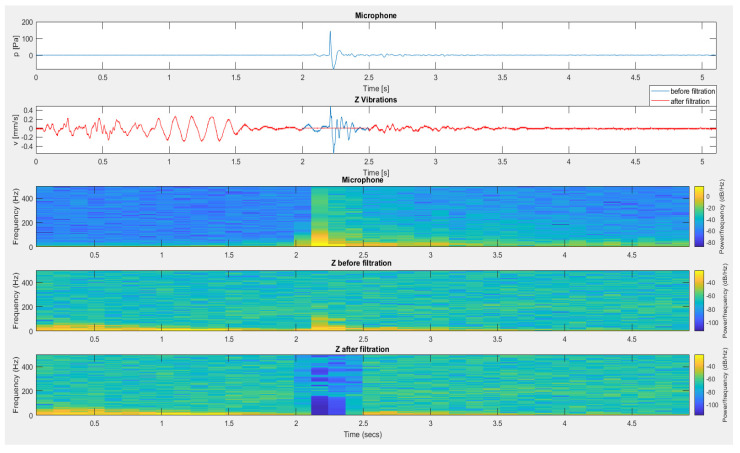
The result of using filtration for the vertical component—firing a series of long-hole blasts.

The last example of filtration application is the recording of signals recorded during measurements in an opencast mine setting when firing a series of long-hole blasts (see Figure 22). Before filtering, a significant impact on the geophone resulting from the propagated airblast wave occurred at approx. 2.2 s. This disturbance resulted in greater vibrations than vibrations propagated by the ground, which, as a consequence, may have disturbed the correct determination of the equation of vibration propagation in the ground. The use of filtration using the airblast microphone signal resulted in the de-noising of this part of the recording.

The presented examples of the use of a microphone signal for airblast pressure measurement to filter the signal from vibration sensors confirmed its practical application and, at the same time, confirmed the possibility of the significant impact of both airblast waves and acoustic waves on vibration velocity sensors (geophones).

## 4. Conclusions

The tests carried out in the reverberation chamber—stimulation with a modulated signal (sine sweep) in the 30–300 Hz range—and explosion of 3 g of pyrotechnic material confirmed the possibility of recording interference in the form of an acoustic wave through a geophone and a microphone for measuring the airblast wave. At the same time, it can be stated that the location of the geophone is very important for interference in the recording of vibration speeds. The most isolated geophone (suspended on the structure) recorded the lowest amplitude values for vibration velocity in all tests. The geophone placed on the mat (acoustic foam) recorded lower values than the sensor placed on the floor, but only for horizontal components, because the vertical component showed similar values. After analyzing the results from the reverberation chamber, it can be stated that noise with very high sound pressure, but without a sudden increase, does not cause a large impact on the sensor itself, but it can cause the formation of a structural acoustic wave that will be recorded by geophones. Thus it will cause signal interference, which will not be mechanical vibrations propagated by the ground. The same phenomenon probably occurred in the case of pyrotechnic material detonation at the training ground [30]. An air acoustic wave caused the formation of a structural wave in the ground and in the structure of the building, and then, as a result of wave penetration, an air acoustic wave inside the object was again formed. This could cause more interference on the internal sensor than the external sensor.

The filtration algorithm presented and the program developed based on it enabled the reduction of interference from seismic signals. Thanks to this, it is possible to select the signal propagated by the substrate, so the conditions presented in the standards [28,29] for assessing the impact of vibrations on building structures will be met.

The presented analyses were performed for the effects induced during the detonation of explosives, but a similar phenomenon may occur during measurements of vibrations induced by traffic, e.g., fast train travel on an uneven track. In addition, it should be remembered that the microphone membranes are stimulated by vibrations transmitted through the ground to the microphone stand (this is observed when determining the frequency performance characteristics of the electrostatic exciter method in accordance with IEC 61094 standard) [40].

In future studies, testing will be expanded to verify other interactions and to refine filtering based on more advanced time–frequency methods that will allow for accurate time correlation with the occurring frequency in the signal. This is due to the fact that both the disturbance and the basic signal can have the same frequency at different times. Therefore, in the presented model, filtering is limited only to the time of interference.
